# Molecular Cloning and Functional Characterization of Three 5-HT Receptor Genes (*HTR1B*, *HTR1E*, and *HTR1F*) in Chickens

**DOI:** 10.3390/genes12060891

**Published:** 2021-06-09

**Authors:** Caiyun Sun, Yang Qiu, Qin Ren, Xiao Zhang, Baolong Cao, Yi Zou, Juan Li, Jiannan Zhang, Yajun Wang

**Affiliations:** Key Laboratory of Bio-Resources and Eco-Environment of Ministry of Education, College of Life Sciences, Sichuan University, Chengdu 610065, China; suncaiyun1237@gmail.com (C.S.); qiuyang1@stu.scu.edu.cn (Y.Q.); renqin@stu.scu.edu.cn (Q.R.); zxzx@stu.scu.edu.cn (X.Z.); cbl-scu@outlook.com (B.C.); 2018141241080@stu.scu.edu.cn (Y.Z.); lijuanscuhk@163.com (J.L.); cdwyjhk@gmail.com (Y.W.)

**Keywords:** chicken, serotonin, serotonin receptors, signal pathway, tissue expression, prolactin

## Abstract

The serotonin (5-hydroxytryptamine, 5-HT) signaling system is involved in a variety of physiological functions, including the control of cognition, reward, learning, memory, and vasoconstriction in vertebrates. Contrary to the extensive studies in the mammalian system, little is known about the molecular characteristics of the avian serotonin signaling network. In this study, we cloned and characterized the full-length cDNA of three serotonin receptor genes (*HTR1B*, *HTR1E* and *HTR1F*) in chicken pituitaries. Synteny analyses indicated that *HTR1B*, *HTR1E* and *HTR1F* were highly conserved across vertebrates. Cell-based luciferase reporter assays showed that the three chicken HTRs were functional, capable of binding their natural ligands (5-HT) or selective agonists (CP94253, BRL54443, and LY344864) and inhibiting intracellular cAMP production in a dose-dependent manner. Moreover, activation of these receptors could stimulate the MAPK/ERK signaling cascade. Quantitative real-time PCR analyses revealed that *HTR1B*, *HTR1E* and *HTR1F* were primarily expressed in various brain regions and the pituitary. In cultured chicken pituitary cells, we found that LY344864 could significantly inhibit the secretion of PRL stimulated by vasoactive intestinal peptide (VIP) or forskolin, revealing that HTR1F might be involved in the release of prolactin in chicken. Our findings provide insights into the molecular mechanism and facilitate a better understanding of the serotonergic modulation via HTR1B, HTR1E and HTR1F in avian species.

## 1. Introduction

5-hydroxytryptamine (5-HT), also known as serotonin, is a bioamine that exerts multiple effects in the peripheral and central nervous system (CNS) [1]. Since its isolation in 1948, 5-HT has been shown to act as a hormone and neurotransmitter in the regulation of various physiological and pathological effects, including psychological disorders, platelet aggregation, vascular tone, hypertension, pulmonary hypertension, intestinal motility, and emesis [2].

The multiple physio-pathological effects of 5-HT are mediated by 5-HT receptors [1], which belong to a phylogenetically ancient receptor family. The 5-HT receptor family consists of at least 14 distinct members and can be divided into seven major classes (HTR1-7) [3,4]. All 5-HT receptors belong to G-protein-coupled receptors [5,6], except HTR3, which is a ligand-gated ion channel receptor. Based on their structural and functional properties, HTR1A, HTR1B, HTR1D, HTR1E and HTR1F were classified as HTR1 members [7]. The amino acid sequences of the five HTR1s share >60% sequence identity within their transmembrane regions [7]. In addition, all HTR1 members show high affinity for 5-HT, and their transduction system is negatively coupled with adenylate cyclase [7]. Among the HTR1 members, HTR1B, HTR1E, and HTR1F are expressed in the CNS and peripheral tissues such as the pituitary, digestive tract and adipose tissue, and play important roles in these tissues in mammals [1]. Studies have shown that rat HTR1B is expressed in the hippocampus, striatum, cerebral cortex, and cerebellum (in particular, Purkinje cells) [8]. Activation of HTR1B is suggested to regulate the release of neurotransmitters related to animal physiology and behaviors, including prolactin secretion, adrenocorticotropic secretion, cortisol and renin secretion, food intake, thermoregulation, sexual and motor behavior [1,3,9,10,11]. HTR1E is abundantly expressed in the human frontal cortex, hippocampus, and olfactory bulb, which are the memory-related centers in the brain, hinting that HTR1E may be involved in the regulation of memory [12]. The full-length cDNA of HTR1F was first cloned in mice, and its pharmacological properties were found to be similar to HTR1E; thus, it is sometimes aliased as HTR1Eβ [13]. Interestingly, HTR1F and HTR1E have a marking difference in their affinity for sumatriptan [7]. Using in situ hybridization, it was discovered that mammalian HTR1F was mainly distributed in various brain regions, including the dorsal raphe nucleus, hippocampus, cerebral cortex, striatum, thalamus, and hypothalamus [14]. In addition, almost all vestibular ganglia, and most trigeminal ganglia of monkeys, showed HTR1F expression in immunohistochemical study [15]. In humans, the HTR1F receptor is considered a target for migraine control [16,17,18] and its effect reportedly occurs in the trigeminal nerve endings around blood vessels [19]. In addition, pancreatic β cells release 5-HT, which regulates the release of glucagon in diabetic mice by activating HTR1F on pancreatic α cells [20].

Similar to mammalian studies, 5-HT regulates a variety of physiological and behavioral responses in avian species. Intraventricular injection of 5-HT reduced the food intake of young chicks and increased their water intake [21]. In addition, injection of 5-HT eight hours before the pre-ovulation period caused ovulation failure in 90% of hens [22]. When broiler pituitaries and hypothalamic were co-cultured, 5-HT and quipazine (a non-selective HTR2 agonist) stimulated the release of PRL and inhibited the release of GH in dose-dependent manners, while the methysergide (a mixed HTR1/2 antagonist) blocked the effect of 5-HT and quipazine on PRL release [23]. Intraperitoneal injection of the HTR1B antagonist GR-127935 in 24-week-old hens for five consecutive days increased the frequency of feather pecking and aggressive behavior in birds [24]. Similarly, brain HTR1B expression was shown to be significantly higher in the high feather pecking group than in the low feather pecking group in White Leghorn chickens [25]. The HTR1F receptor is also reported to be involved in avian early embryonic development [26]. Contrary to the extensive studies of HTR1B, HTR1E, and HTR1F in mammalian species, little is known regarding their structure and functions in non-mammalian vertebrates, including birds. In our study, using the chicken as an animal model, we identified the full-length cDNA sequences, motifs, and studied the signaling properties of HTR1B, HTR1E, and HTR1F. We also characterized their expression profile in various tissues and these findings suggested their potential roles in the regulation of pituitary PRL secretion. Our findings facilitate a better understanding of HTR family genes and provide the molecular basis to elucidate the physiological roles of HTR1B, HTR1E, and HTR1F in birds.

## 2. Materials and Methods

### 2.1. Chemicals, Primers, Peptides, and Antibodies

All chemicals, including 5-HT, CP94253, BRL54443 and LY344864, were purchased from Sigma-Aldrich (St. Louis, MO, USA), unless stated otherwise. Restriction enzymes were obtained from Takara Biotechnology Co (Dalian, China). Antibodies used in this study include phospho-p44/42 MAPK (Erk1/2) (Thr202/Tyr204) rabbit mAb (1:1000, #9101), phospho-CREB (Ser133) (87G3) rabbit mAb (1:1000, #9198), β-actin (ACTB, 13E5) rabbit mAb (1:2000, #4970) and anti-rabbit IgG, HRP-linked antibody (1:5000, #7074), all of which were purchased from Cell Signaling Technology (Danvers, MA, USA). All primers used in this study were synthesized by Beijing Qingke Biotechnology Company (Chengdu, China), the primers were listed in Supplemental Table S1.

### 2.2. Animals and Tissues

Adult chickens or chicks (Lohmann layer) used in this study were purchased from a local commercial company. Chicken tissues, including the spinal cord, telencephalon, midbrain, cerebellum, hindbrain, hypothalamus, anterior pituitary, gizzard, proventriculus, duodenum, jejunum, ileum, cecum, colon, crop, heart, liver, spleen, lung, kidney, muscle, testes, ovary, skin, pancreas, and subcutaneous fat, were collected from the euthanized animals and frozen in liquid nitrogen immediately. All samples were stored at −80 °C until further analysis. Anterior pituitaries collected from 6 adult chickens were used to measure the relative mRNA levels of *HTR1B*, *HTR1E*, and *HTR1F* in the caudal lobe (Ca) and cephalic lobe (Ce). All animal experimental protocols used in this study were approved by the Animal Ethics Committee of College of Life Sciences, Sichuan University, China, and the assurance number is 20210308008 (8 March 2021).

### 2.3. Reverse Transcription (RT) and Quantitative Real-Time PCR

As described in our previous study [27,28], total RNA from chicken tissues was purified using RNAzol (Molecular Research Center) and dissolved in Diethylpyrocarbonate (DEPC)-treated H_2_O. These RNA samples were then reverse transcribed by Moloney murine leukemia virus (M-MLV) reverse transcriptase (Takara) and were either subjected to PCR amplification of target genes from chicken pituitaries, or quantitative real-time PCR assay (qRT-PCR). In brief, oligodeoxythymide (0.5 μg) and total RNA (2 μg) were mixed in a total volume of 5 μL, incubated at 70 °C for 10 min, and cooled at 4 °C for 2 min. Then, the first step buffer, 0.5 mM each of deoxynucleotide triphosphate and 100 U MMLV reverse transcriptase were added into the reaction mix with a total volume of 10 μL. Reverse transcription (RT) was performed at 42 °C for 90 min.

qRT-PCR was conducted on the CFX96 Real-Time PCR Detection System (Bio-Rad, Hercules, CA, USA) to examine the mRNA levels of target genes in chicken tissues, as previously established [27,29]. qRT-PCR was performed in a volume of 20 μL containing 0.5 μL RT product, 1 × PCR buffer, 0.2 mM each dNTP, 2.5 mM MgCl_2_, 0.2 mM each primer, 0.5 U Taq DNA polymerase (Takara), and 1 μL EvaGreen (Biotium Inc., Hayward, CA, USA), under the following conditions: 2 min at 94 °C denaturation, followed by 40 cycles (20 s at 94 °C, 15 s at 62 °C, and 20 s at 72 °C) of reaction, ending with a 10 min extension at 72 °C. To assess the specificity of PCR amplification, melting curve analysis and agarose gel electrophoresis were performed at the end of the reaction to confirm that a specific PCR band was amplified.

### 2.4. Phylogenetic Analysis

The predicted chicken HTR1 protein sequences were aligned with orthologs from other vertebrates using BioEdit software, version 7.2.5 [30]. Phylogenetic analysis was computed using MEGA7 software [31], in which the phylogenetic tree was constructed with the neighbor-joining method, and confidence was estimated with 1000 bootstrap replicates. The NCBI accession numbers of the protein sequences used in the present study are shown in Supplemental Table S3.

### 2.5. Rapid Amplification of 5′- and 3′-cDNA Ends (RACE) and Construction of Plasmids

To characterize the 5′-untranslated region (UTR) and 3′-UTR region of chicken *HTR1B, HTR1E, and HTR1F*, rapid amplification of 5′- or 3′-cDNA ends (RACE) was performed using SMART^TM^ RACE cDNA Amplification Kit (Clontech, Palo Alto, CA, USA) according to the manufacturer’s instructions. The amplified PCR products were cloned into pTA2 vector (TOYOBO, Osaka, Japan) for sequencing. Based on the full-length cDNA sequences of *HTR1B* (accession number: NM_001172781), *HTR1E* (accession number: MK139005) and *HTR1F* (accession number: MK139006), their complete open reading frames (ORFs) were amplified by PCR from the chicken pituitary cDNA and cloned into the pcDNA3.1(+) eukaryotic expression vector (Invitrogen, Carlsbad, CA, USA).

### 2.6. Functional Characterization of Chicken HTR1 Receptors

According to our previously established methods [29,32,33], chicken HTR1B or HTR1E or HTR1F was transiently expressed in human embryonic kidney 293 (HEK293) cells and treated by 5-HT (10^−11^ to 10^−7^ M) or HTR1 agonists (CP94253, BRL54443 or LY344864, 10^−11^ to 10^−5^ M) for six hours. The receptor-activated signaling pathways were then examined by pGL3-CRE-luciferase (capable of monitoring cAMP/PKA signaling pathways) and pGL4-SRE-luciferase reporter systems (capable of monitoring MAPK/ERK signaling pathways). Luminescence was measured by a multimode microplate reader (TriStar LB941, EG&G Berthold, Bad Wildbad, Germany) according to the manufacturer’s instruction.

### 2.7. Western Blot

HEK293 cells transfected with HTR1B, HTR1E or HTR1F expression plasmid were cultured on a 24-well plate at 37 °C and then treated with 5-HT for 10 min. Then, cells were lysed with 1 × passive lysis buffer (Promega). The phosphorylated ERK1/2 (pERK1/2) and CREB (pCREB) levels in cell lysates were examined by Western blot, as described in our previous studies [29,34].

### 2.8. Effect of 5-HT, CP94253, BRL54443 and LY344864 on PRL Secretion in Cultured Chick Pituitary Cells

As described in the previous experiment [35,36], anterior pituitaries were collected from 3-week-old male or female chicks; then, digested by 0.25% trypsin at 37 °C for 30 min. The dispersed pituitary cells were then cultured at a density of 5 × 10^5^ cells/well on Corning^®^ CellBIND^®^ 48-well plates (Corning, Lowell, MA, USA) with culture medium 199 (M199, Gibco, Grand Island, NY, USA) containing 10% FBS (Gibco) at 37 °C with 5% CO_2_. After 18 h of culture, the culture medium was removed, and cells were treated with 120 μL M-199 containing various concentration of 5-HT (0.1 μM) or CP94253 (10 μM) or BRL54443 (10 μM) or LY344864 (10 μM) in the presence of VIP (5 nM) or forskolin (FK, 2 μM). After 6 h of treatment, the culture medium was collected for measurement of PRL secretion, and then pituitary cells were lysed with 1 × passive lysis buffer (Promega) and lysates were collected to examine intracellular PRL and β-actin levels. The protein samples were electrophoresed and transferred to the membrane, and then the standard procedures were used for protein detection. The PRL rabbit polyclonal antibody (1:500) against recombinant full-length chicken PRL were prepared in our laboratory and used to detect the PRL protein levels in our previous study [35,36].

### 2.9. Data Analysis

Relative PRL levels were calculated as the ratio to the intracellular β-actin level and then expressed as a percentage of control or VIP or forskolin treatment groups. The relative mRNA level of each gene was first calculated as the ratio to that of β-actin and then expressed as fold change compared with the respective control. GraphPad Prism 7 (GraphPad Software Inc, San Diego, California, USA) was used for statistical analysis. Data were analyzed by the unpaired Student’s *t*-test (for two groups) or by one-way ANOVA, followed by the Dunnett’s test (for comparing treatment groups with control). Data are presented as mean ± SEM. To validate our results, all experiments were repeated at least 2 or 3 times. *p* ≤ 0.05 (*), *p* ≤ 0.01 (**) or *p* ≤ 0.001 (***) indicates that the data are statistically significant.

## 3. Results

### 3.1. Molecular Cloning of HTR1B, HTR1E, and HTR1F in Chickens

**Figure 1:**
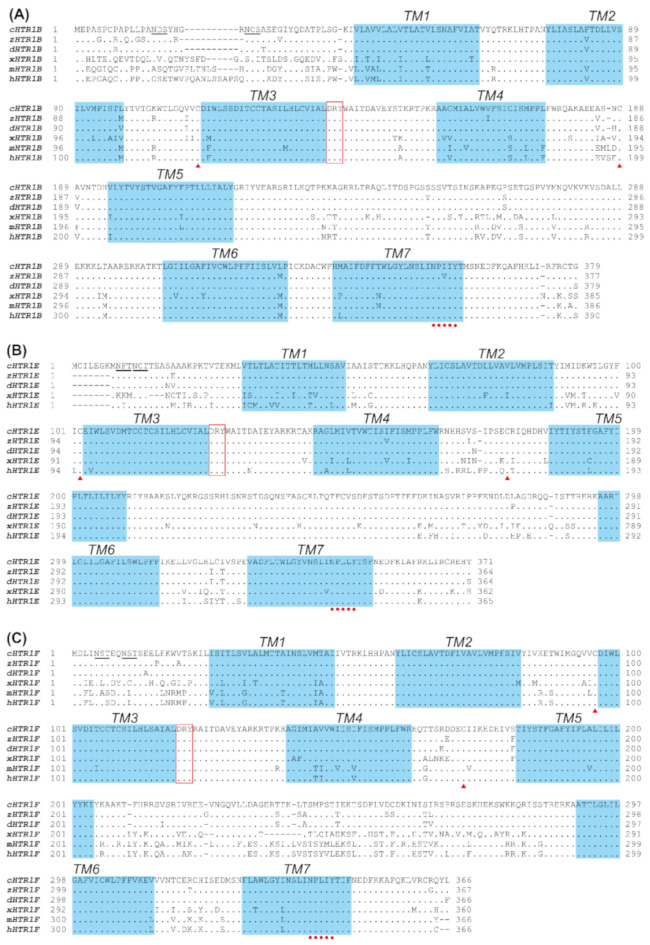
Amino acid sequence alignment of chicken HTR1B, HTR1E, and HTR1F with that of other species. (**A**) Amino acid alignment of chicken HTR1B (cHTR1B, XP_015140120.1) with that of zebra finch (zHTR1B, XP_002190169.2), ducks (dHTR1B, XP_005017926.2), *Xenopus tropicalis* (xHTR1B, XP_002936251.2), mice (mHTR1B, NP_034612.1) and humans (hHTR1B, NP_000854.1). (**B**) Amino acid alignment of chicken HTR1E (cHTR1E, MK139005) with that of zebra finch (zHTR1E, XP_032603125.1), ducks (dHTR1E, XP_005013102.1), *X. tropicalis* (xHTR1E, XP_002933964.1) and humans (hHTR1E, NP_000856.1). (**C**) Amino acid alignment of chicken HTR1F (cHTR1F, MK139006) with that of zebra finch (zHTR1F, XP_002191608.1), ducks (dHTR1F, XP_005017708.2), *X. tropicalis* (xHTR1F, XP_002931817.1), mice (mHTR1F, NP_032336.1) and humans (hHTR1F, NP_000857.1). The conserved DRY motif is boxed in red, and TM1-7 (seven transmembrane domains) are shaded in blue. The red dots indicate the conserved NPXXY motif. The red triangles indicate the conserved cysteine residues for disulfide bond formation. The predicted N-glycosylation sites are underlined. ‘.’ represents identical amino acid, and ‘-’ indicates a gap in the sequence alignment.

According to the predicted chicken *HTR1B* sequence (XM_015284634.2), *HTR1E* sequence (XM_015284707.2) and *HTR1F* sequence (XM_004938334.3) in GenBank, the full-length cDNA sequences of the three HTR1s were cloned from the chicken pituitary by RACE-PCR and RT-PCR (Figure 1 and Supplemental Table S1). The coding regions of *HTR1B, HTR1E, and HTR1F* are 1140 bp, 1116 bp and 1101 bp in length, respectively, and were predicted to encode receptors of 379, 371 and 366 amino acid (a.a.) residues, respectively (Figure 1).

The chicken HTR1B, HTR1E, and HTR1F amino acid sequences were compared with orthologs from other species (Supplemental Table S2). Our results indicate that the amino acid sequence of chicken HTR1B shows a high degree of sequence identity with HTR1B from other species, including zebra finches (95%), ducks (97%), *Xenopus tropicalis* (81%), mice (86%) and humans (83%). The amino acid sequence of chicken HTR1E shows a high degree of sequence identity with HTR1E of zebra finches (95%), ducks (97%), *X. tropicalis* (81%), and humans (83%). The amino acid sequence of chicken HTR1F shows a high degree of sequence identity with HTR1F of zebra finches (95%), ducks (97%), *X. tropicalis* (81%), mice (80%) and humans (83%) (Figure 1).

Like HTR1s from other vertebrate species, chicken HTR1B, HTR1E, and HTR1F all contain seven hydrophobic transmembrane domains (TMD1–7), a conserved ‘DRY’ motif at the C-terminal of the third transmembrane domain (TMD3) essential for receptor function, two cysteine residues for the formation of a disulfide bond. Moreover, two potential N-glycosylation sites were also noted at the N-terminus of HTR1B, HTR1E, and HTR1F (Figure 1).

### 3.2. Phylogenetic Analysis and Synteny Analysis of HTR1B, HTR1E, and HTR1F in Vertebrates

**Figure 2:**
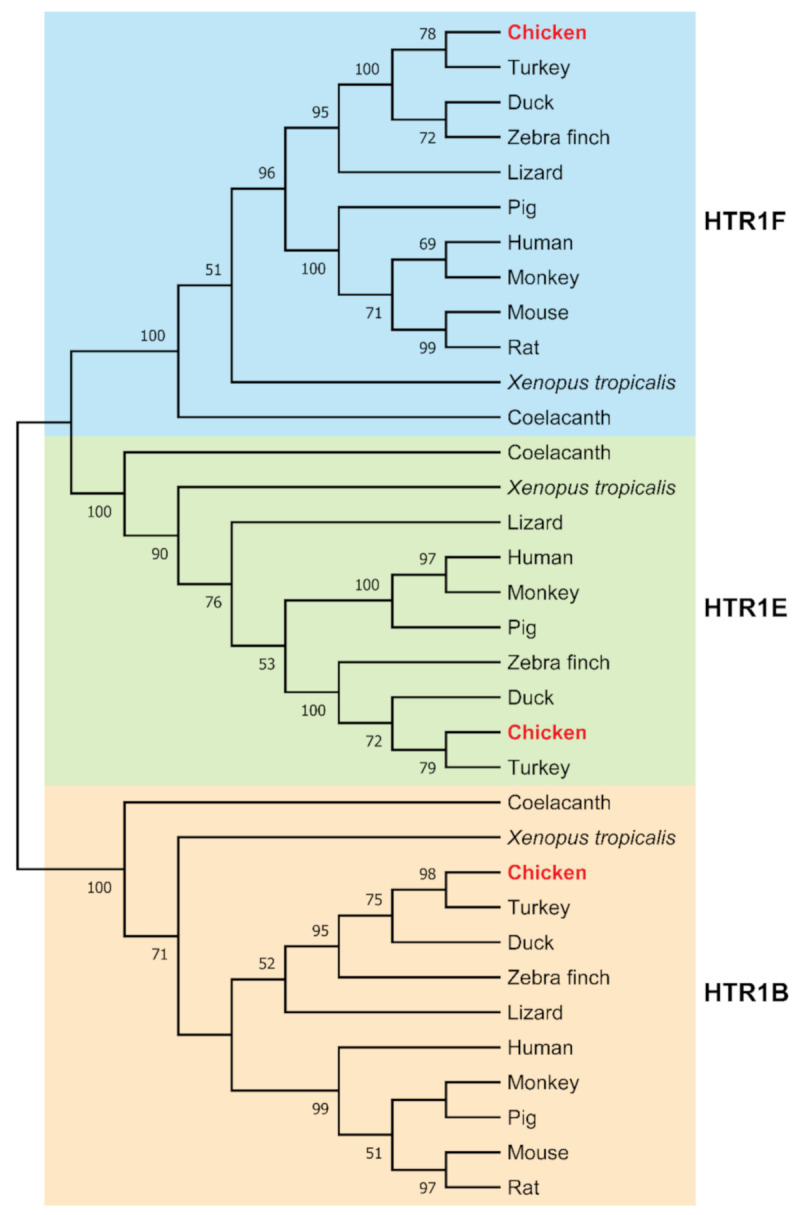
The phylogenetic tree of *HTR1B*, *HTR1E*, and *HTR1F*. Based on 1000 bootstrap replicates, the neighbor-joining method was used to construct the phylogenetic tree. The bootstrap value is indicated near the branch point.

To investigate the evolutionary relationship of HTR1B, HTR1E, and HTR1F receptors in vertebrates, the amino acid sequences of HTR1B, HTR1E, and HTR1F receptors of different species were subjected to phylogenetic analysis. As shown in Figure 2, the receptor sequences form three clusters, with the chicken ortholog each found with its corresponding orthologs as expected. Notably, HTR1E cluster is closer to HTR1F cluster than to HTR1B cluster in vertebrates (Figure 2).

**Figure 3:**
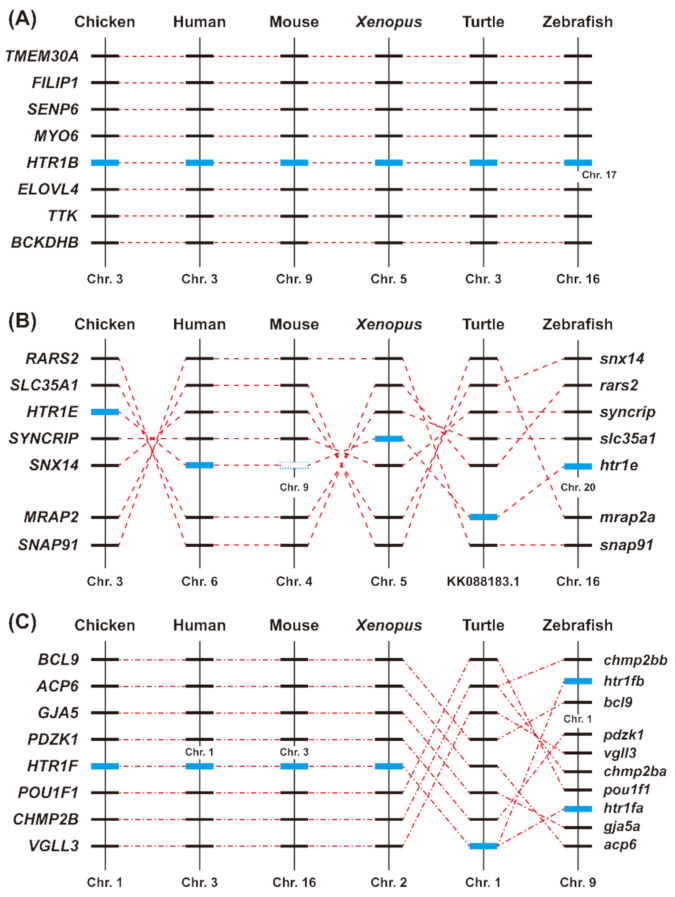
Synteny analysis of *HTR1B*, *HTR1E*, and *HTR1F* in chickens and other vertebrates. *HTR1B* (**A**), *HTR1E* (**B**), and *HTR1F* (**C**) are located in three syntenic regions conserved in vertebrate species, including chickens, humans, mice, *X. tropicalis*, painted turtles, and zebrafish. Blue boxes denote the genes of interest, while dotted lines indicate the syntenic genes identified in these species. Chr—chromosome.

To further elucidate whether chicken *HTR1s* are orthologous to *HTR1s* in humans and other vertebrates, synteny analysis was performed by searching the conserved neighboring genes of *HTR1s* in the genomes of humans, mice, *X. tropicalis*, turtles, and zebrafish using the Ensembl database (v95.01) and the Genomicus genome browser [37]. As shown in Figure 3A, a conserved gene cluster, including *HTR1B* and its adjacent genes, was identified in genomes of nearly all species studied, indicating that the synteny of *HTR1B* is highly conserved across vertebrates. In the present study, the synteny of *HTR1E* gene is also conserved in chickens, humans, *X. tropicalis*, turtles, and zebrafish. Interestingly, *HTR1E* gene was likely lost in mice during evolution (Figure 3B). Similar to *HTR1B*, *HTR1F* and its adjacent genes are also conserved in chickens, humans, mice, *X. tropicalis*, turtles, and zebrafish, indicating that *HTR1F* is highly conserved across vertebrates (Figure 3C). Interestingly, there are two copies of *htr1f* in zebrafish, named *htr1fa* and *htr1fb*.

### 3.3. Functional Characterization of HTR1B, HTR1E, and HTR1F in Cultured HEK293 Cells

**Figure 4:**
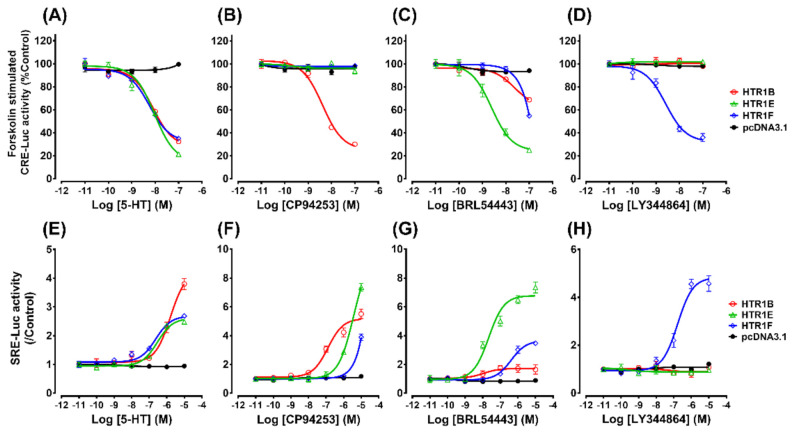
Luciferase activities of HEK293 cells expressing chicken HTR1B, HTR1E, and HTR1F in response to various concentrations of 5-HT (or receptor-selective agonists) treatments. HEK293 cells were transiently co-transfected with HTR1 expression plasmid and pGL3-CRE/pGL4-SRE luciferase reporter construct, and subjected to ligand treatment for 6 h before measurement. Effects of 5-HT (**A**), CP94253 (**B**), BRL54443 (**C**), and LY344864 (**D**) (10^−11^–10^−7^ M, 6 h) on forskolin (2 μM)-stimulated luciferase activity of HEK293 cells expressing chicken HTR1B, HTR1E, and HTR1F, monitored by a pGL3-CRE luciferase reporter system. Effects of 5-HT (**E**), CP94253 (**F**), BRL54443 (**G**), and LY344864 (**H**) (10^−11^–10^−5^ M, 6 h) on luciferase activity of HEK293 cells expressing chicken HTR1B, HTR1E, and HTR1F, monitored by a pGL4-SRE luciferase reporter system. HEK293 cells co-transfected with empty pcDNA3.1(+) and luciferase reporter construct were used as a control. Each data point represents the mean ± SEM of 3 replicates.

Luciferase reporter assays were performed to determine the signaling properties of HTR1B, HTR1E, and HTR1F using different ligands, including 5-HT (natural ligand), CP94253 (a selective HTR1B agonist) [38,39,40], BRL54443 (a selective HTR1E agonist) [41,42,43] and LY344864 (a selective HTR1F agonist) [44,45,46] respectively. Each receptor was transiently expressed in HEK293 cells and treated with 5-HT or a selective agonist (CP94253, BRL54443 or LY344864) in the presence of 2 μM forskolin (a diterpene activator of adenylate cyclase). Using a pGL3-CRE-luciferase reporter system, the receptor-mediated inhibition of the cAMP/PKA signaling pathway was subsequently monitored as in our previous studies [32]. As shown in Figure 4A–D, HTR1B could inhibit forskolin-stimulated luciferase activity via activation of 5-HT (EC_50_ = 8.0 nM) or CP94253 (EC_50_ = 4.0 nM); similarly, HTR1E could inhibit forskolin-stimulated luciferase activity via activation of 5-HT (EC_50_ = 9.3 nM) or BRL54443 (EC_50_ = 2.4 nM); HTR1F also could inhibit forskolin-stimulated luciferase activity via activation of 5-HT (EC_50_ = 5.7 nM) or LY344864 (EC_50_ = 1.8 nM) (Table 1). Taken together, these results indicated that chicken HTR1B, HTR1E, and HTR1F are functional receptors, and their activation down-regulates the intracellular cAMP level in a dose-dependent manner. In addition, CP94253, BRL54443 and LY344864 were shown to be selective agonists for chicken HTR1B, HTR1E, and HTR1F, respectively. Their EC_50_ values are slightly lower than that of the natural ligand, 5-HT.

Using a pGL4-SRE-luciferase reporter system (Figure 4E–H), 5-HT (EC_50_ = 876.9 nM) and CP94253 (EC_50_ = 274.0 nM) could stimulate luciferase activities of HEK293 cells expressing HTR1B. Similarly, 5-HT (EC_50_ = 150.6 nM) and BRL54443 (EC_50_ = 28.8 nM) could stimulate luciferase activities of HEK293 cells expressing HTR1E. Both 5-HT (EC_50_ = 531.2 nM) and LY344864 (EC_50_ = 270.3 nM) could stimulate luciferase activities of HEK293 cells expressing HTR1F. Although the corresponding EC_50_ values of 5-HT are higher than those of CP94253, BRL54443, and LY344864 (Table 1), chicken HTR1B, HTR1E, and HTR1F activation were shown to stimulate the MAPK/ERK signaling cascade.

**Figure 5:**
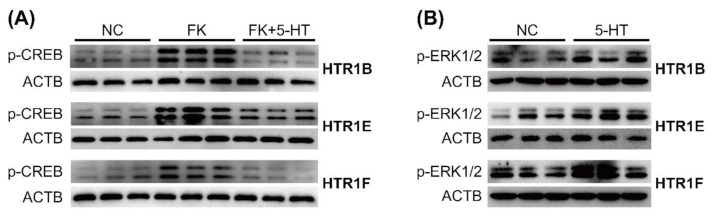
Western blot showing 5-HT treatment (100 nM, 10min) can inhibit the forskolin (FK) (2 μM)-stimulated phosphorylation levels of CREB (p-CREB) in HEK293 cells expressing chicken HTR1B, HTR1E, and HTR1F (**A**), and 5-HT treatment (10 μM, 10 min) can enhance phosphorylation of ERK1/2 (p-ERK1/2) in HEK293 cells expressing chicken HTR1B, HTR1E, and HTR1F (**B**).

In addition, Western blots were performed to elucidate these signaling properties of chicken HTR1B, HTR1E, and HTR1F. As shown in Figure 5A, 5-HT treatment (100 nM, 10 min) could inhibit forskolin (2 μM)-stimulated CREB phosphorylation in HEK293 cells expressing HTR1B, HTR1E, and HTR1F, respectively. Similarly, 5-HT (10 μM, 10 min) treatment could enhance ERK1/2 (44/42 kDa) phosphorylation in HEK293 cells expressing HTR1B, HTR1E, and HTR1F, respectively (Figure 5B). These findings further supported the functional coupling of the three receptors to the cAMP/PKA and MAPK/ERK signaling cascade, in agreement with previous luciferase assay results.

### 3.4. Tissue Distribution of HTR1B, HTR1E, and HTR1F in Chickens

**Figure 6:**
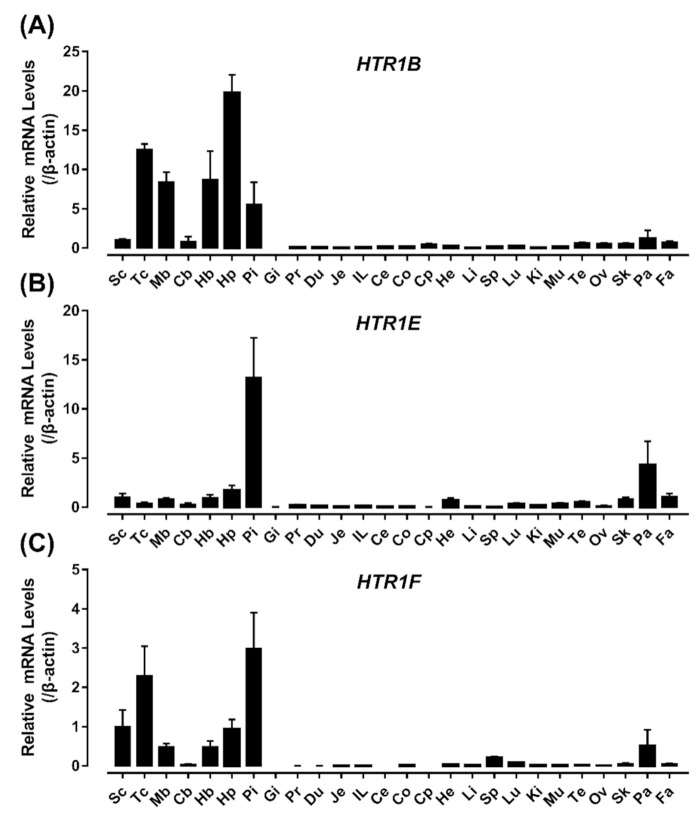
qRT-PCR detection of *HTR1B* (**A**), *HTR1E* (**B**) and *HTR1F* (**C**) in various adult chicken tissues, including the spinal cord (Sc), telencephalon (Tc), midbrain (Mb), cerebellum (Cb), hindbrain (Hb), hypothalamus (Hp), anterior pituitary (Pi), gizzards (Gi), proventriculus (Pr), duodenum (Du), jejunum (Je), ileum (IL), cecum (Ce), colon (Co), crop (Cp), heart (He), liver (Li), spleen (Sp), lung (Lu), kidney (Ki), muscle (Mu), testes (Te), ovary (Ov), skin (Sk), pancreas (Pa), and subcutaneous fat (Fa). β-actin was used as an internal control. The mRNA levels of receptors in chicken tissues were expressed as fold difference compared with that in the spinal cord (Sc). Each data point represents the mean ± SEM of 6 individual adult chickens (3 males and 3 females) (*n* = 6).

To reveal the physiological roles of HTR1B, HTR1E, and HTR1F in chickens, qRT-PCR was performed to examine their mRNA expression in adult chicken tissues, including the spinal cord, anterior pituitary, proventriculus, gizzards, duodenum, jejunum, ileum, cecal, colon, heart, kidneys, liver, lung, muscle, ovary, testes, spleen, pancreas, subcutaneous fat, skin, crop, and various brain regions (e.g., telencephalon, midbrain, cerebellum, hindbrain, and hypothalamus). As shown in Figure 6, *HTR1B* is extensively expressed in various brain regions (e.g., telencephalon, midbrain, hindbrain, and hypothalamus) with high abundance in the anterior pituitary, moderate expression in the pancreas, skin, and subcutaneous fat, and weak expression in other tissues examined, but not in gizzards (Figure 6A). *HTR1E* mRNA is highly expressed in the pituitary, moderately expressed in the pancreas, heart, spinal cord, skin, subcutaneous fat, and various brain regions (e.g., hypothalamus, midbrain, and hindbrain) (Figure 6B). In the present study, *HTR1F* mRNA is highly expressed in the anterior pituitary and various brain regions (e.g., telencephalon, hypothalamus, midbrain, and hindbrain) (Figure 6C).

**Figure 7:**
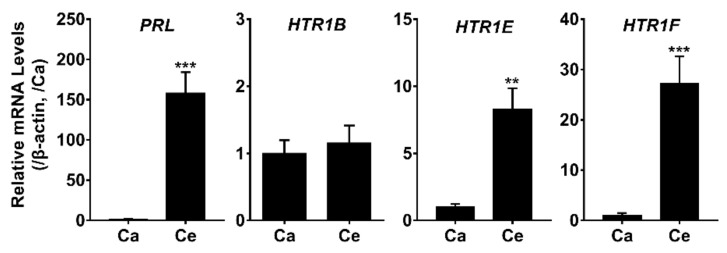
qRT-PCR assay of *PRL*, *HTR1B, HTR1E, and HTR1F* mRNA expression in the cephalic lobe (Ce) and caudal lobe (Ca) of adult chicken anterior pituitary, β-actin was used as an internal control. The mRNA levels of target genes were normalized by that of β-actin and expressed as the fold difference compared with that of the caudal lobe (Ca), **, *p* ≤ 0.01 vs. Ca; ***, *p* ≤ 0.001 vs. Ca. Each data point represents the mean ± SEM of six replicates (*n* = 6).

In the present study, all three receptor transcripts were shown to be highly expressed in the anterior pituitary, which functions as the master gland controlling the release of a wide range of hormones; thus, prompting us to further probe their spatial localization within the anterior pituitary. The relative mRNA levels of *PRL*, *HTR1B, HTR1E, and HTR1F* in the cephalic (Ce) and caudal lobe (Ca) were examined by qRT-PCR. As shown in Figure 7, both *HTR1E* and *HTR1F* mRNA are exclusively expressed in the cephalic lobe of chicken anterior pituitaries, in which PRL also has a high expression.

### 3.5. LY344864 Inhibits VIP-Induced PRL Secretion in Cultured Chicken Pituitary Cells

**Figure 8:**
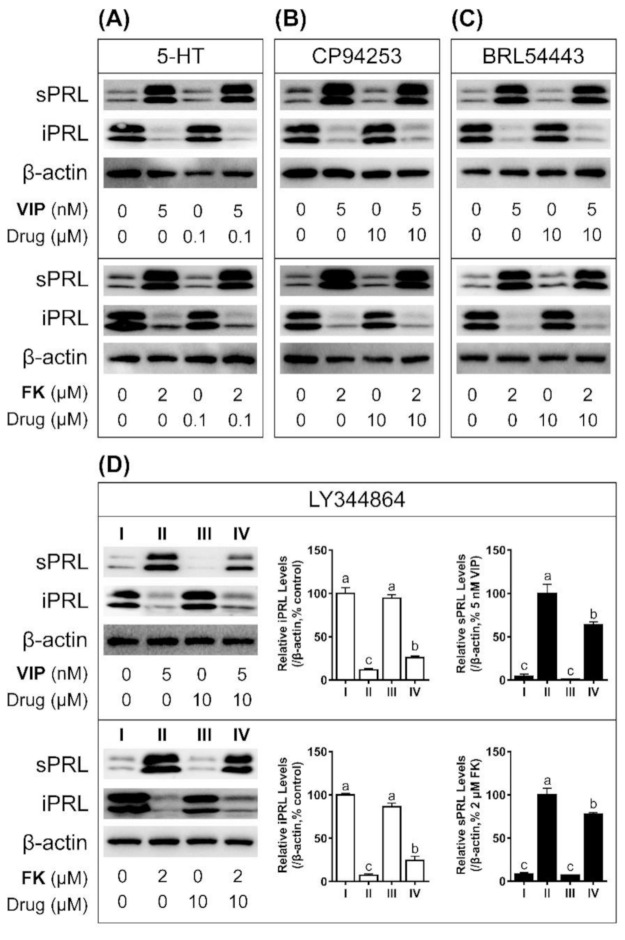
Western blot showing the effects of various selective agonists ((**A**): 5-HT; (**B**): CP94253; (**C**): BRL54443; (**D**): LY344864) on VIP (5 nM) or forskolin (FK, 2 μM)-stimulated cPRL secretion in cultured chicken pituitary cells. sPRL represents secretory PRL, and iPRL represents intracellular PRL. Protein levels were semi-quantified by densitometry, β-actin was used as an internal control. For the sPRL analysis, the 5 nM VIP or 2 μM FK treated group was regarded as 100%, while for the iPRL analysis, the control group (without any drug treatment) was regarded as 100%. Different letters indicate statistical difference between two groups (*p* ≤ 0.01), as determined by one-way ANOVA and a Tukey’s test. Each data point represents the mean ± SEM of three replicates (*n* = 3). The cPRL bands were observed at 24 kDa and 27 kDa (glycosylated form), as reported in our previous studies [36].

To investigate the potential roles of chicken *HTR1B, HTR1E, and HTR1F* in pituitary function, Western blot was performed to examine the effects of 5-HT, CP94253, BRL54443 and LY34486 on VIP (5 nM)-induced PRL secretion in cultured chicken pituitary cells. As shown in Figure 8D, LY344864 (10 μM, 6 h) could significantly inhibit VIP-stimulated PRL secretion in cultured pituitary cells. However, 5-HT (0.1 μM, 6 h), CP94253 (10 μM, 6 h), and BRL54443 (10 μM, 6 h) showed no significant effect on VIP-stimulated PRL secretion (Figure 8A–C).

To clarify whether the cAMP/PKA signaling pathway is involved in pituitary PRL release, we examined the effect of 5-HT, CP94253, BRL54443 and LY344864 on PRL secretion in the presence of forskolin (2 μM). As described in previous studies [36], forskolin (2 μM, 6 h) stimulated PRL secretion in cultured chicken pituitary cells. LY344864 (10 μM, 6 h) treatment could significantly inhibit PRL secretion in this study (Figure 8D). However, 5-HT (0.1 μM, 6 h), CP94253 (10 μM, 6 h) and BRL54443 (10 μM, 6 h) showed no significant effect on PRL release (Figure 8A–C). Our study showed that HTR1F could inhibit pituitary PRL secretion by inhibiting the cAMP/PKA signaling pathway, but HTR1B and HTR1E could not.

## 4. Discussion

In this study, HTR1B, HTR1E, and HTR1F were cloned from the chicken pituitary. The functional study demonstrated that HTR1B, HTR1E, and HTR1F are functional and can bind to 5-HT and activate downstream signaling pathways. qRT-PCR assays revealed that *HTR1B, HTR1E, and HTR1F* mRNA are widely expressed in chicken tissues with high abundance in the brain and pituitary. Moreover, the selective HTR1F agonist LY344864 has been shown to inhibit VIP-induced PRL secretion in cultured chicken pituitary cells.

In this study, the full-length cDNA for the three chicken *HTR1s* (*HTR1B, HTR1E, and HTR1F*) were cloned from the chicken pituitary. Sequence alignment showed that these three receptors shared more than 80% amino acid identity with that of mammals (e.g., humans, mice), reptiles (e.g., painted turtles) and amphibians (e.g., *X. tropicalis*), suggesting *HTR1s* (*HTR1B, HTR1E, and HTR1F*) were conserved in vertebrates (Figure 1). All HTR1B, HTR1E, and HTR1F sequences identified in mammals, reptiles, fish, and birds were clustered into three clades in the phylogenetic analysis (Figure 2). Similarly, synteny analysis (Figure 3) also supported that chicken *HTR1B, HTR1E, and HTR1F* are orthologous to their counterparts in other vertebrates. Interestingly, mouse *HTR1E* could not be found in our search in the genomic database and via synteny analysis, suggesting that this might have been lost during evolution in this lineage (Figure 3B). This also concurs with the report that *HTR1E* has not been cloned from mice or rats [47]. On the other hand, two copies of *HTR1F* genes, named *htr1fa* and *htr1fb*, were found in the zebrafish genome (Figure 3C). It is speculated that zebrafish often had more than one ortholog of human genes during evolution [48], which leads to the possibility of the existence of two copies of the *htr1f* gene in zebrafish, suggesting that the evolutionary process of *HTR1E* and *HTR1F* in vertebrates is complex and their physiological roles may be variable, but it may need further investigation.

In this study, pGL3-CRE-luciferase and pGL4-SRE-luciferase reporter systems were employed to detect whether HTR1B, HTR1E, and HTR1F could be activated by 5-HT, CP94253, BRL54443, and LY344864 in HEK293 cells. Our results demonstrated that HTR1B can be activated by 5-HT and CP94253, and in turn can inhibit the cAMP/PKA signaling pathway and activate the MAPK/ERK signaling pathway (Figure 4 and Figure 5). Compared with 5-HT, CP94253 showed a higher potency on HTR1B, as shown in Table 1. Consistent with studies in mammals, as an inhibitory G-protein-coupled receptor, chicken HTR1B preferentially coupled with Gi/o to inhibit cAMP formation [49,50]. In humans, HTR1B stimulated with 5-HT efficiently activates ERK with an EC_50_ value of 2.4 nM [51]. The stimulation of mouse HTR1B also leads to the activation of the extracellular signal-regulated protein kinase 2 (ERK2) in transfected Chinese hamster ovary (CHO) cells [52]. Among the three selective agonists examined in this study, CP94253 showed a high affinity for HTR1B only in the physiological dose (4 nM) vs. the other two receptors, suggesting that CP94253 is a selective HTR1B receptor agonist in chicken as well.

In our study, chicken HTR1E could be activated by 5-HT and BRL54443 in a dose-dependent manner (Figure 4) and could inhibit the cAMP/PKA signaling pathway and stimulate the MAPK/ERK signaling pathway. Our findings are consistent with previous reports in mammalian species [43,53]. In mammals, 5-HT binds to the guinea pig HTR1E receptor to stimulate [^35^S]GTPγS in a dose-dependent manner with an EC_50_ of 13.6 ± 1.92 nM, which is similar to the EC_50_ of the human HTR1E receptor (13.7 ± 1.78 nM) [54]. Interestingly, the tryptophan-associated agonist BRL54443 was demonstrated in our study to be a selective agonist for the chicken HTR1E receptor (EC_50_ = 2.4 nM), with an affinity 100 times higher than that of HTR1F. This contrasts reports in humans, in which BRL54443 showed a similar affinity to HTR1E and HTR1F [53,55]. Our findings revealed that BRL54443 is more selective for chicken HTR1E than the HTR1F; this difference in pharmacological effect on the two receptors between birds and humans warrants further studies, especially how their structures may influence ligand selectivity.

In HEK293 cells expressing chicken HTR1F, both 5-HT and LY344864 could inhibit forskolin-stimulated cAMP accumulation in a concentration-related manner. Our finding indicates that the chicken HTR1F receptor is coupled to the MAPK/ERK signaling pathway (Figure 4 and Figure 5). In addition, LY344864 was found to be a selective agonist for chicken HTR1F in our study, similar to previous in vitro and in vivo reports in mammals [56]. In particular, LY344864 exhibits a high affinity for HTR1F with pKd of 8.2 which is ~100-fold higher than that for HTR1A, HTR1B, HTR1D, and HTR1E in mammals [57].

In this study, qRT-PCR was employed to detect the expression profile of *HTR1B, HTR1E, and HTR1F* in adult chicken tissues. Our current finding on chicken *HTR1B* mRNA expression being higher in the telencephalon, midbrain, hindbrain, hypothalamus, and pituitary gland than other peripheral tissues is consistent with the reports in mammals, in which *HTR1B* expressed highly in the hypothalamus and pituitary [58,59,60,61]. Nevertheless, it is worth noting that *HTR1B* mRNA expression in the cerebellum is relatively low in chickens (Figure 6A), which differs from the situation in mammals [58,60]. In zebrafish, *HTR1B* in situ labeling was also not detected in the cerebellum [62]. This could have occurred because of the diversity of brain architecture between mammals and birds/fish. The expression of *HTR1B* in thalamic nuclei has been implicated in controlling sensory maps in both the somatosensory and visual systems [63]. Consistent with the *HTR1B* distribution in the telencephalon of chicks [64], the high *HTR1B* expression in the brain region suggested the conserved modulatory function of HTR1B in chicken.

As reported in mammals, *HTR1E* mRNA is mainly expressed in the pituitary and widely expressed in the brain regions, including the telencephalon, midbrain, cerebellum, hindbrain, and hypothalamus in chicken (Figure 6B). However, the cellular localization of HTR1E in the chicken brain regions need further investigations. In guinea pigs, immunohistochemical and pharmacological studies demonstrated that HTR1E is widely expressed in the brain and cerebral vascular system, such as the olfactory bulb and dentate gyrus [65]. In humans and monkeys, the expression of *HTR1E* mRNA has been detected in the cerebral cortex, including the entorhinal cortex, caudate nucleus, and putamen [66]. In the present study, *HTR1E* mRNA was found to be highly expressed in the pancreas, revealing its potential role in regulating the secretion of insulin and glucagon similar to that in mammals [67,68,69].

Our qRT-PCR analyses showed that *HTR1F* is also highly expressed in various brain regions (e.g., the telencephalon, midbrain, hindbrain, and hypothalamus), spinal cord and pituitary (Figure 6C), which is consistent with the report in mammals [14,70,71]. A previous in situ hybridization study revealed that *HTR1F* mRNA is localized in the hippocampus, cortex (especially the cortex and entorhinal cortex) and dorsal cortical nucleus of mice and guinea pigs [66]. Studies in humans also strongly support the hypothesis that *HTR1F* plays a major role in migraines and is a potential anti-migraine drug target [18,72]. Members of the 5-hydroxytryptamine receptor (HTR) family, including HTR1F, have been identified in goose ovaries, and these receptors were speculated to affect egg production by regulating ovarian metabolic function [73]. Further studies are required to investigate the distribution and function of HTR1F in avian.

The consistently high expression of *HTR1B, HTR1E, and HTR1F* in the pituitary in our current study urged us to investigate their physiological roles further in these cells. As shown in Figure 8D, LY344864 could significantly inhibit the VIP- or forskolin-induced PRL secretion in cultured primary chicken pituitary cells. Prolactin is mainly secreted by lactotrophs in the anterior pituitary. It regulates multiple physiological processes in birds, such as reproductive behaviors, egg-laying, metabolism, development, and regulation of the hypothalamic–pituitary–gonadal axis [74,75]. In chickens, PRL secretion is controlled by VIP, thyrotropin-releasing hormone (TRH), arginine vasotocin (AVT) and negatively by dopamine (DA) and neuropeptide W (NPW) [36,76]. This study represents the first to prove that LY344864 is a novel non-peptide inhibitory factor on the pituitary PRL secretion via HTR1F in chickens. In mammals, the effect of 5-HT via different receptors on prolactin is controversial. Some studies reported that 8-OH-DPAT (a HTR1A agonist) has a weak inhibitory effect on PRL in perfused rat pituitary cells [77,78]. In the study by Papageorgiou and Denef, HTR4 was shown to be involved in the release of PRL from the pituitary since the HTR4 antagonist GR-113808 could completely antagonize the release of PRL stimulated by 5-HT in rat pituitary cells [77]. Ketanserin (a HTR2 antagonist) was shown to inhibit the release of PRL stimulated by 5-HT or TRH [79]. In the study [80], the intraperitoneal injection of methysergide (a HTR1 and HTR2 antagonist), ketanserin, LY53857 (a HTR2 antagonist), ICS205-930 (a HTR3 antagonist) and GR38032F (a HTR3 antagonist) could reduce the 5-HT, 5-hydroxytryptophan (5-HTP) or fluoxetine (5-HT reuptake inhibitor)-stimulated serum PRL level. The complex and diverse modulatory function of the 5-HT system may be related to physiological difference in the reproductive system/behaviors between mammals and birds, which is worthy of further investigation.

In summary, the full-length cDNA of the three chicken 5-HT receptor genes (*HTR1B*, *HTR1E*, and *HTR1F*) were first reported in the present study. Sequence alignment showed that the three genes showed high sequence identity with their counterparts in other vertebrates. Moreover, synteny analysis revealed that *HTR1B, HTR1E, and HTR1F* are highly conserved among vertebrates and supported previous findings that chicken receptors are orthologous to the known receptor groups. Functional studies demonstrated that HTR1B, HTR1E, and HTR1F are functional and responsive to 5-HT and various selective agonists CP94253, BRL54443 and LY344864, respectively. Together with the enriched expression of the three receptors in the pituitary, the observation that LY344864 can partially inhibit PRL secretion in cultured chicken pituitary cells suggest that HTR1F is likely involved in regulating the release of the chicken pituitary PRL. Our findings represent the first key step in establishing the molecular basis for the better understanding of the serotonergic modulation network in avian species.

## Figures and Tables

**Table 1:** EC_50_ values of 5-HT, CP94253, BRL54443 and LY344864 in activating different signaling pathways in HEK293 cells expressing chicken HTR1B, HTR1E or HTR1F.

EC_50_ Values (nM)				
Drug	5-HT	CP94253	BRL54443	LY344864
cAMP/PKA signaling pathway				
HTR1B	8.0	4.0	/	/
HTR1E	9.3	/	2.4	/
HTR1F	5.7	/	/	1.8
MAPK/ERK signaling pathway				
HTR1B	>800 ^a^	>200 ^a^	/	/
HTR1E	>100 ^a^	/	28.8	/
HTR1F	>500 ^a^	/	/	>200 ^a^

‘/’ means that the EC_50_ values could not be calculated based on the experimental data. ^a^ Indicates that the EC_50_ value was roughly estimated based on the experimental data.

## Supplementary Materials

The following are available online at https://www.mdpi.com/article/10.3390/genes12060891/s1, Table S1: Primers used in this study, Table S2. Lists of genes and their GenBank accession numbers used in amino acid sequence alignment, Table S3. Lists of genes and their GenBank accession numbers used to generate the phylogenetic tree in this study.

## Abbreviations

5-HT: serotonin; 5-HTP: 5-hydroxytryptophan; AC: adenylate cyclase; CNS: central nervous system; ERK: extracellular signaling-regulated kinase; HTR1B: serotonin receptor 1B; HTR1E: serotonin receptor 1E; HTR1F: serotonin receptor 1F; HEK293: human embryonic kidney 293 cells; MAPK: mitogen-activated protein kinase; PRL: prolactin; PKA: protein kinase A; qRT-PCR: Quantitative Real-Time PCR; VIP: vasoactive intestinal polypeptide.
