# Supplementary material for: Molecular Cloning and Functional Characterization of Three 5-HT Receptor Genes (HTR1B, HTR1E, and HTR1F) in Chickens
Source: Genes (Basel). 2021 Jun 9;12(6):891. doi: 10.3390/genes12060891 (PMC8230051; doi:10.3390/genes12060891)
Supplement: Supplementary file 1 [file genes-12-00891-s001.zip › genes-1227986-supplementary.pdf]

Molecular Cloning and Functional Characterization of Three 5-HT Receptor Genes (HTR1B, HTR1E, and HTR1F) in Chickens

Caiyun Sun, Yang Qiu, Qin Ren, Xiao Zhang, Baolong Cao, Yi Zou, Juan Li, Jiannan Zhang* and Yajun Wang

Supplementary Materials

Table S1. Primers used in this study.

Gene	Primer name	Primer sequences (5'-3')
[a]*Primer for cloning the coding regions*		
cHTR1B	U1	*CGGAATTCATGGAGCCGGCGAGCCCC*
	L1	*GCTCTAGATCAGCCTGTGCAGCGGAA*
cHTR1E	U1	*GGGGTACCATGTGCATCCTGGAGGGG*
	L1	*GCTCTAGATTAAGTATGTTCTCTACA*
cHTR1F	U1	*CGGAATTCAACATGGATTTAATAAAC*
	L1	*GCTCTAGACTCTTAAAGATATTGCCT*
[a]*Primer for quantitative RT-PCR assays*		
cHTR1B	U1	*GGGGAAAAGACTGACTCGGG*
	L1	*GTCTTTGTCGCTTTCCGCTC*
cHTR1E	U1	*AGCAAGTGACCACCTTTGGG*
	L1	*AAGTAGCTGCTTTGGGTAGCA*
cHTR1F	U1	*CACGTCACATTCGCTCCTCT*
	L1	*GTCCTTCCTTTGCTACCCCG*
cPRL	U1	*CCATTCTCTGGAAGGCTGTAGA*
	L1	*CTGGAGTCCTCATCAGCGAGTT*
β-actin	U1	*CCCAGACATCAGGGTGTGATG*
	L1	*GTTGGTGACAATACCGTGTTCAAT*

Table S2. Lists of genes and their GenBank accession numbers used in amino acid sequence alignment.

	Chicken	Zebra finch	Duck	*Xenopus tropicalis*	Mouse	Human
HTR1B	XP_015140120.1	XP_002190169.2	XP_005017926.2	XP_002936251.2	NP_034612.1	NP_000854.1
HTR1E	MK139005	XP_032603125.1	XP_005013102.1	XP_002933964.1	-	NP_000856.1
HTR1F	MK139006	XP_002191608.1	XP_005017708.2	XP_002931817.1	NP_032336.1	NP_000857.1

Table S3. Lists of genes and their GenBank accession numbers used to generate the phylogenetic tree in this study.

Gene names	Species	GenBank accession numbers
	Chicken	XP_015140120.1
HTR1B	Turkey	XP_010725582.1
	Duck	XP_005017926.2
	Zebra finch	XP_002190169.2
	Lizard	XP_034966577.1
	Pig	NP_999463.1
	Human	NP_000854.1
	Monkey	XP_008004603.1
	Mouse	NP_034612.1
	Rat	NP_071561.1
	Xenopus tropicalis	XP_002936251.2
	Coelacanth	XP_006012936.1
HTR1E	Chicken	MK139005
	Turkey	XP_019468658.1
	Duck	XP_005013102.1
	Zebra finch	XP_032603125.1
	Lizard	XP_034965333.1
	Pig	XP_013848119.2
	Human	NP_000856.1
	Monkey	XP_008004718.1
	Xenopus tropicalis	XP_002933964.1
	Coelacanth	XP_006009258.1
HTR1F	Chicken	MK139006
	Turkey	XP_003202888.1
	Duck	XP_005017708.2
	Zebra finch	XP_002191608.1
	Lizard	XP_034971820.1
	Pig	NP_999266.1
	Human	NP_000857.1
	Monkey	XP_037859109.1
	Mouse	NP_032336.1
	Rat	NP_068629.2
	Xenopus tropicalis	XP_002931817.1
	Coelacanth	XP_006011232.1
